# The endogenous neuropeptide calcitonin gene-related peptide after spontaneous subarachnoid hemorrhage–A potential psychoactive prognostic serum biomarker of pain-associated neuropsychological symptoms

**DOI:** 10.3389/fneur.2022.889213

**Published:** 2022-07-28

**Authors:** Elisabeth Bründl, Martin Proescholdt, Eva-Maria Störr, Petra Schödel, Sylvia Bele, Florian Zeman, Christoph Hohenberger, Martin Kieninger, Nils Ole Schmidt, Karl-Michael Schebesch

**Affiliations:** ^1^Department of Neurosurgery, University Medical Center Regensburg, Regensburg, Germany; ^2^Section Neurosurgery, Department of Orthopedics, Trauma and Hand Surgery, Medical Center St. Elisabeth, Straubing, Germany; ^3^Center for Clinical Studies, University Medical Center Regensburg, Regensburg, Germany; ^4^Department of Anesthesiology, University Medical Center Regensburg, Regensburg, Germany

**Keywords:** biomarker, calcitonin gene-related peptide (CGRP), cognitive impairment, health-related quality of life (hrQoL), neuropeptide, pain, somatoform syndrome, spontaneous subarachnoid hemorrhage

## Abstract

**Background:**

The pronociceptive neuromediator calcitonin gene-related peptide (CGRP) is associated with pain transmission and modulation. After spontaneous subarachnoid hemorrhage (sSAH), the vasodilatory CGRP is excessively released into cerebrospinal fluid (CSF) and serum and modulates psycho-behavioral function. In CSF, the hypersecretion of CGRP subacutely after good-grade sSAH was significantly correlated with an impaired health-related quality of life (hrQoL). Now, we prospectively analyzed the treatment-specific differences in the secretion of endogenous CGRP into serum after good-grade sSAH and its impact on hrQoL.

**Methods:**

Twenty-six consecutive patients (f:m = 13:8; mean age 50.6 years) with good-grade sSAH were enrolled (drop out *n* = 5): *n* = 9 underwent endovascular aneurysm occlusion, *n* = 6 microsurgery, and *n* = 6 patients with perimesencephalic SAH received standardized intensive medical care. Plasma was drawn daily from day 1 to 10, at 3 weeks, and at the 6-month follow-up (FU). CGRP levels were determined with competitive enzyme immunoassay in duplicate serum samples. All patients underwent neuropsychological self-report assessment after the onset of sSAH (t_1_: day 11–35) and at the FU (t_2_).

**Results:**

During the first 10 days, the mean CGRP levels in serum (0.470 ± 0.10 ng/ml) were significantly lower than the previously analyzed mean CGRP values in CSF (0.662 ± 0.173; *p* = 0.0001). The mean serum CGRP levels within the first 10 days did not differ significantly from the values at 3 weeks (*p* = 0.304). At 6 months, the mean serum CGRP value (0.429 ± 0.121 ng/ml) was significantly lower compared to 3 weeks (*p* = 0.010) and compared to the first 10 days (*p* = 0.026). Higher mean serum CGRP levels at 3 weeks (*p* = 0.001) and at 6 months (*p* = 0.005) correlated with a significantly poorer performance in the item pain, and, at 3 weeks, with a higher symptom burden regarding somatoform syndrome (*p* = 0.001) at t_2_.

**Conclusion:**

Our study reveals the first insight into the serum levels of endogenous CGRP in good-grade sSAH patients with regard to hrQoL. In serum, upregulated CGRP levels at 3 weeks and 6 months seem to be associated with a poorer mid-term hrQoL in terms of pain. In migraineurs, CGRP receptor antagonists have proven clinical efficacy. Our findings corroborate the potential capacity of CGRP in pain processing.

## Highlights

- Impairment in health-related quality of life (hrQoL) after treatment of spontaneous subarachnoid hemorrhage (sSAH) is common but underreported.
- Calcitonin gene-related peptide (CGRP), a potent vasoactive and pronociceptive neuromediator, is involved in pain transmission and modulation of psycho-behavioral function.
- This study is the first to correlate endogenous plasma CGRP with mid-term hrQoL outcome in good-grade sSAH.
- During the first 10 days after sSAH, the mean CGRP levels in serum range significantly lower than in CSF and serum CGRP levels decrease significantly over 6 months.
- Upregulated plasma CGRP levels at 3 weeks and 6 months after sSAH seem to be significantly associated with pain and somatoform syndrome.
- As is the case with migraine, after sSAH, upregulated plasma CGRP is suggested to be involved in the pathogenesis of pain and pain processing.

## Introduction

Aneurysmal subarachnoid hemorrhage (aSAH) is a serious and highly complex neurovascular insult demanding specialized multidisciplinary therapy concepts during the acute phase, rehabilitation, socio-professional reintegration, and follow-up (FU) care. Over the past few decades, advanced neurovascular treatment strategies, early aneurysm repair, and individualized neuro-intensive care management have not only accounted for declining trends in case-fatality but also for an improvement in functional outcome (1). However, the regained functional independence contrasts with an appallingly low rate of return to work ranging from 70.8% (2) to <50% (3) to merely 3% (4) of the patients surviving spontaneous subarachnoid hemorrhage (sSAH). In up to 50 % of the sSAH patients, the bleeding is accompanied by subtle, yet substantial neuropsychological impairment, fatigue, emotional dysbalance, and somatic problems like bodily pain, which in turn seriously affect the patients' health-related quality of life (hrQoL) (3, 5). Profound literature on the longitudinal course of sSAH-associated bodily pain, especially headache, and its management is sparse (2). Persistent severe to worst headache, refractory to analgesics (6), is reported by more than 90% of the sSAH patients and represents the second-leading cause for 30-day hospital readmission after sSAH (7). Long-term FU data indicates that–in case of inadequate treatment (6, 8)–sSAH-associated headache may persist for 2–9 years after the hemorrhage (8–10). The pathogenesis of headache after aneurysm rupture is deemed to be multifactorially mediated, but the exact mechanisms remain elusive (11).

In light of the risen clinical awareness of the long-term disabilitiy in cognition and day-to-day functioning, researchers and clinicians strive for the establishment of reliable, clinically relevant biomarkers with a predictive capacity for neurocognitive outcome and hrQoL. Recently, the endogenous 37-amino acid neuropeptide Calcitonin gene-related peptide (CGRP) (12) has gained paramount interest as a potential vasoactive and psychoactive biomarker in cerebrospinal fluid (CSF) and serum after sSAH. CGRP is stored in sensory perivascular nerve fibers which arise from the trigeminal ganglia of Gasseri and acts as a major, highly potent microvascular vasodilator (13). In aSAH, the proven secretion of CGRP into CSF (14, 15) was suggested to be associated with a potential neuroprotective effect by preventing cerebral vasospasm (CV) and cerebral ischemia (15) but with a deleterious effect on the hrQoL in the acute phase (16). Beyond, the neuromodulator CGRP (17) attested a crucial involvement in various neurobehavioral processes (18) like in inflammatory and neuropathic pain (19), and, by unalterable, massive relaxation of cerebral arteries, in migraine (20). In 2018, specifically designed drugs acting antagonistically on CGRP or on the CGRP receptor have ushered in a new era in migraine therapy for acute relief of migraine and effective prevention of migraine attacks (21).

Up to now, no data is yet available on the impact of good-grade sSAH on the intrinsic release of CGRP into serum, on its temporal dynamics, and its pathophysiological interactions with higher integrated behavior. Our study aims to determine whether CGRP could prove to be a useful predictive surrogate parameter in serum for the self-reported hrQoL up to 6 months after sSAH.

## Patients and methods

The study protocol, the prospective liquid biobanking, and the clinical database of this prospective single-center investigation were approved by the Local Institutional Ethics Committee (reference number 06-179). The study follows the principles of the Declaration of Helsinki.

### Patient population

The cohort has been reported previously (16, 22, 23). A total of 26 consecutive patients, treated for acute non-traumatic, angiographically confirmed aneurysmal or non-aneurysmal perimesencephalic SAH (pSAH) at our University Medical Center between February 2013 and May 2016, fulfilled the beforehand specified eligibility criteria of being native German speakers, aged 18–75 years, who had been admitted to hospital within 48 h of ictus in prognostically favorable, good to moderate neurological condition, defined as a World Federation of Neurological Surgeons (WFNS) score (24) and Hunt and Hess (HH) grading (25) of 1–4, respectively, and an initial Glasgow Coma Scale (GCS) of ≥9. Written informed consent was obtained from each individual. In all patients, the diagnosis of sSAH was based on cerebral computed tomography (CT) and a four-vessel digital subtraction angiography (DSA) to visualize the location and morphology of the underlying ruptured aneurysm and to differentiate aneurysmal from perimesencephalic hemorrhages, respectively. Within the first 72 h after the onset of sSAH, all patients received an external ventricular drain (EVD) due to a radiologically confirmed acute occlusive hydrocephalus. Treatment consisted either of endovascular aneurysm occlusion (EV group) or microsurgical clipping (MS group) for aSAH patients, followed by standardized therapy in the intensive care unit (ICU) (pSAH group). Our standardized microsurgical and endovascular procedure protocols (26) and our ICU standard operating protocol (27) have been described elsewhere.

The clinical database comprised all demographic, radiological, and neurological variables, comorbidities, non-/invasive procedures, complications, comprehensive pharmacological screening (at discharge and at the 6-month FU), and outcome grading [Glasgow Outcome Scale (GOS) (28) and modified Ranking Scale (mRS) (29)].

### Neuropsychological self-report assessment

Subacutely (between day 11 and 35; t_1_) and 6 months (t_2_) after the onset of sSAH, all patients completed two well-established generic self-report measures of physical and mental health each: The ICD-10-Symptom-Rating questionnaire (ISR) (30) serves as a score for symptom burden and the German version of the 36-Item Short Form Health Survey (SF-36) (31) represents a performance score.

### Laboratory procedures

Within the 10-day period after the onset of sSAH, plasma was drawn directly from the arterial line once daily. At 3 weeks and at the 6 month-FU, blood was collected by venipuncture. Immediately subsequent to sampling, the samples were centrifuged at 1,200 rounds per minute for 10 min, and the supernatants were aliquoted and stored at−80°C until further use. The samples were thawed, aliquoted (1 ml) with columns [STRATA C18-E (55 μm, 70 A) 200 mg/3 ml-columns, Phoenix Pharmaceuticals Inc., Burlingame, USA], purified, evaporated on a vacuum concentrator (Christ RVC 2-25 CD plus; Osterode am Harz, Germany), and dissolved in 250 μl assay-buffer resulting in a fourfold concentration. CGRP levels were measured in duplicate purified serum samples using a competitive enzyme immunoassay (EIA; Phoenix Pharmaceuticals Inc., Burlingame, CA, USA). The cerebral exposure to the endogenously released CGRP into serum over time is expressed as ng/ml.

### Statistical analysis

Continuous data and neuropsychological test results are presented as mean ± standard deviation (SD) and range (minimum to maximum) and categorical data as frequency counts.

*Neuropsychological assessment:* Changes over time within each group were analyzed with a paired *t-*test. Differences between groups at postinterventional assessment were analyzed with an analysis of variance (ANOVA) followed by Fisher's LSD *post-hoc* pairwise comparisons.

*Correlation of CGRP with neuropsychological assessment:* Regression analyses were conducted for correlations of mean CGRP levels with the hrQoL test scores. Changes over time within each group were analyzed with a paired *t*-test. Intragroup variances (correlations between hrQoL test scores and clinical variables) were analyzed using an analysis of variance (Bartlett's test for equal variances). Statistical analysis was conducted according to Stata procedures (Stata Version 14.2; Stata Corp. College Station, TX, USA).

A *p* < 0.05 was considered statistically significant. A biostatistician was involved in the design and realization of the statistical evaluation.

## Results

### Demographics and descriptive statistics

**Figure 1:**
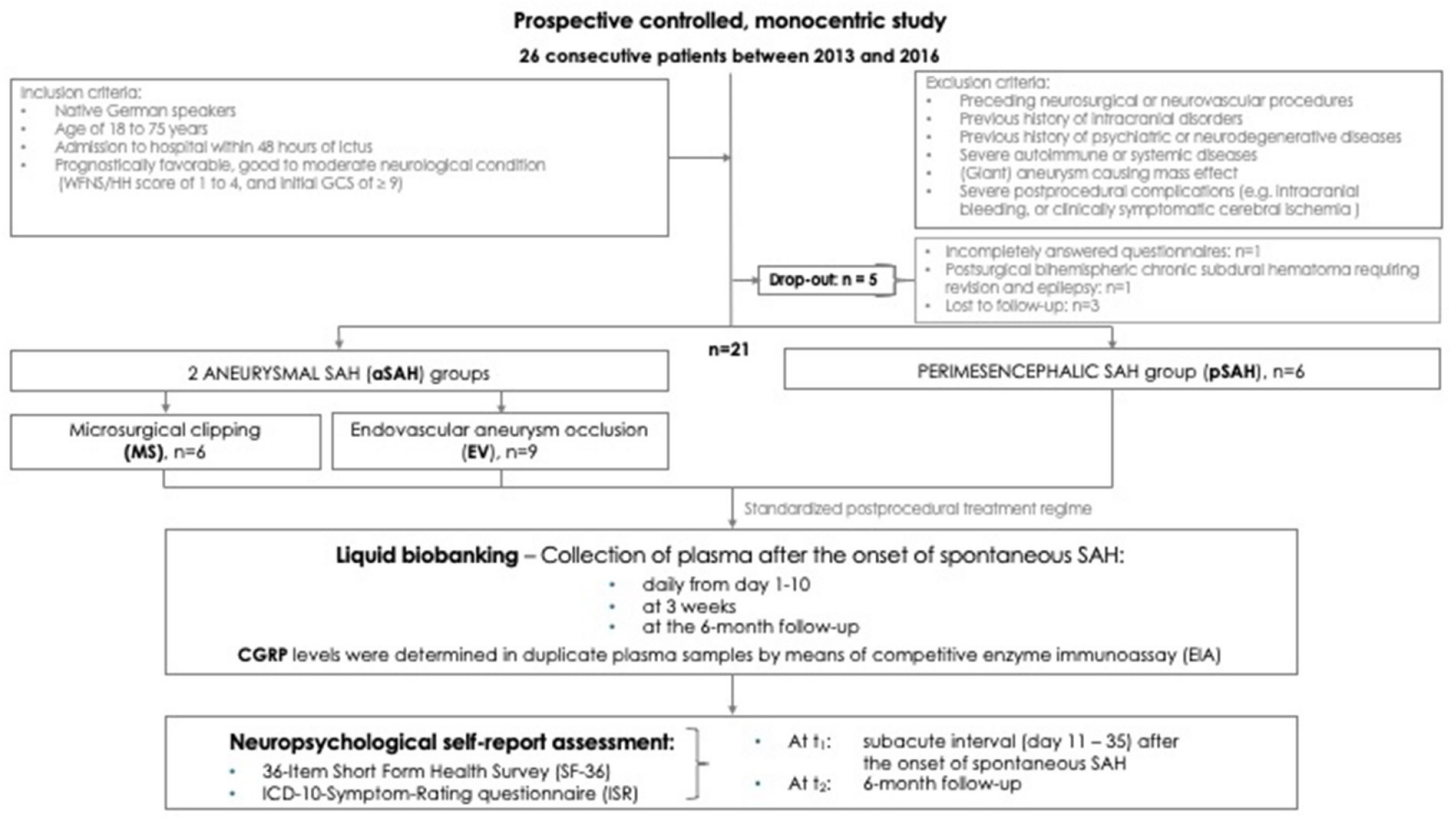
Flowchart. Study design and reasons for exclusion of potentially eligible cases.

A total of *N* = 160 patients with sSAH were treated during the observation period. In accordance with our strict selection criteria, depicted in the Flowchart (Figure 1), five patients were excluded from analysis. Thus, a total of 21 patients (8 men, 13 women) of a mean age of 50.6 years (range 27–72 years) with good-grade sSAH was assigned to statistical evaluation. Four aSAH patients, initially presenting with HH grade III (14%) or IV (5%), neurologically improved immediately after insertion of an EVD following hospital admission (i.e., HH I or HH II). Thus, the poorer HH score was obviously related to acute occlusive hydrocephalus. With this qualification, we consider the term “good-grade” sSAH patients as appropriate for our cohort. An aneurysmal bleeding source in the anterior (*n* = 12) or posterior circulation was detected in 15 patients (EV *n* = 9: coil *n* = 5, stent-assisted coil *n* = 2; balloon-assisted coil *n* = 1; flow diverter *n* = 1). No patient developed serious procedure-related complications like, for example, periprocedural aneurysm rupture, procedure-related blood transfusion, postprocedural onset of a new neurological deficit, or clinically symptomatic CV-related stroke. Functional outcome was stable or even improved over the 6-month FU. None of these patients required decompressive craniectomy, and, until FU, no late rebleedings or mortalities had occurred. Each individual was able to complete both of our surveys, the ISR and the SF-36, within 10–15 min, and without any signs of (mental or physical) fatigue during the testing, neither at t_1_ nor at t_2_. Comprehensive information on the baseline data including the aneurysm site, GCS, HH, WFNS and Fisher score, procedure variables, medication, and outcome grading is provided by Table 1.

**Table 1:** Demographic and clinical characteristics of patients.

**Patient characteristics**	**Study population**		
	**MS**	**EV**	**pSAH**
Number of patients [*n*]	6	9	6
Male to female ratio	2: 4	3: 6	3: 3
Age [years], mean ± SD	42.0 ± 13.2	50.1 ± 9.3	59.8 ± 7.4
(range)	(27–61)	(30–59)	(53–72)
Calcitonin gene-related peptide in serum			
Level within the first 10 days [ng/ml], mean ± SD	0.535 ± 0.094	0.453 ± 0.135	0.435 ± 0.069
Level at 3 weeks [ng/ml], mean ± SD	0.474 ± 0.134	0.465 ± 0.193	0.554 ± 0.151
Level at 6 months [ng/ml], mean ± SD	0.429 ± 0.111	0.420 ± 0.114	0.442 ± 0.160
Calcitonin gene-related peptide in CSF			
Level within the first 10 days [ng/ml], mean ± SD	0.626 ± 0.155	0.698 ± 0.178	0.645 ± 0.201
Site of aneurysm			
Anterior circulation (ruptured) [n]	8 (6)	7 (6)	1 (0)^a^
Posterior circulation (ruptured) [n]	0 (0)	3 (3)	-
	8	10	1^a^
Side of aneurysm [*n*]			
Left	3	2	1^a^
Right	4	2	–
Side of pterional approach			
Dominant-side	3	–	–
Non-dominant side	3	–	–
Initial neurological status			
GCS [*n*]			
15	1	5	4
14	4	2	1
13	1	0	1
11	0	1	0
9	0	1	0
Hunt and Hess score [*n*]			
0	0	0	0
I	2	5	2
II	2	2	4
III	2	1	0
IV	0	1	0
V	0	0	0
WFNS score [*n*]			
1	1	5	4
2	4	2	2
3	1	0	0
4	0	2	0
5	0	0	0
Fisher score [*n*]			
1	0	0	0
2	0	0	0
3	2	2	2
4	4	7	4
External ventricular drainage			
Implantation after the onset of SAH [d], mean ± SD	0.5 ± 0.6	0.4 ± 0.7	0.5 ± 0.6
(range)	(0–1)	(0–2)	(0–1)
*In situ* [d], mean ± SD	15.8 ± 4.9	15.1 ± 7.7	12.0 ± 5.0
(range)	(9–23)	(9–30)	(5–18)
Replacement [*n*]	0	2	1
CSF infection [*n*]	1	4	2
Permanent ventriculoperitoneal shunt placement [*n*]	0	2	2
Implantation after the onset of SAH [d], mean ± SD	-	23.5 ± 0.7	29.5 ± 6.4
(range)	-	(23–24)	(25–34)
Vasospasm [*n*]			
DIND	1	1	0
TCD	6	7	3
DSA	0	2	1
Multimodal neuromonitoring	1	1	0
Intraarterial cerebral spasmolysis^a^	0	2	0
Treatment-associated lesions on CT scan [*n*]			
Circumscribed ischemia^b^	2	2	0
Brain edema	3	1	0
Bleeding along the EVD entry route	1	0	1
Hygroma	0	0	1^c^
Medication at discharge / at FU			
Anticonvulsive drugs	1/1	0/1	0/0
Benzodiazepines	0/0	1/0	0/0
Antidepressants	0/0	3/1	1/1
Neuroleptics	0/0	0/0	0/0
Opioid	2/0	1/1	2/0
Nicotine patches	0/1	0/1	0/1
Antihypertensive drugs	4/3	4/6	3/3
Thyroid medication	3/3	2/2	0/0
Antiplatelet agents	0/0	5^*^/3	0/0
No medication	0/2	0/1	0/2
Neurological status at discharge / at FU			
GOS [*n*]			
5	4/5	7/7	5/6
4	0/0	1/2	1/0
3	2/1	1/0	0/0
mRS [*n*]			
0	4/2	7/3	4/5
1	0/3	0/3	2/1
2	1/0	1/3	0/0
3	0/1	0/0	0/0
4	1/0	1/0	0 /0
Patients with new neurological deficit [*n*]			
At discharge	0	1	0
At FU	1	3	0
Employment status at FU [*n*]			
Returned to work	1	2	1
Attending vocational integration programs	0	2	0
On sick leave	1	3	0
Unemployed	1	1	0
Unemployed before and after the onset of SAH	0	1	0
Retired at the onset of SAH	0	0	1
No information provided	3	0	4

*MS, microsurgical clipping group; EV, endovascular treatment group; pSAH, perimesencephalic subarachnoid hemorrhage group; SD, standard deviation; CSF, cerebrospinal fluid; AUC, area under the curve; ng, nanograms; ml, milliliter; h, hours; GCS, Glasgow Coma Scale; WFNS, World Federation of Neurosurgical Societies; d, days; SAH, subarachnoid hemorrhage; DIND, delayed ischemic neurological deficit; TCD, transcranial Doppler ultrasound; DSA, digital subtraction angiography;*

a *Transient placement of transfemoral microcatheters for local intraarterial cerebral spasmolysis (n = 1 continuous nimodipine infusion for 3 and 5 days; n = 1 single shot infusion of milrinone) to reverse severe cerebral vasospasm*.

b *Clinically silent ischemia, either in the section of the caudate nucleus after unintended clipping of the recurrent artery of Heubner or diffusely in the periventricular white matter;*

c *Conservatively treated bi-hemispheric hygroma due to CSF overdrainage via the EVD; CT scan, computerized axial tomography scan; EVD, external ventricular drainage; FU, 6-month follow-up; GOS, Glasgow Outcome Scale; mRS, modified Rankin Scale*.

* *p < 0.05*.

The presented sSAH cohort exclusively encompassed patients with severe radiological grades of sSAH (Fisher score 3 or 4) and, consecutively, is imbalanced in terms of the overestimation of the true hydrocephalus rate after sSAH. This is all the more remarkable given the inclusion of 6 patients with pSAH. Only four patients, however, required permanent ventriculoperitoneal shunting. Statistical intergroup comparisons yielded no significant differences except for a higher number of middle cerebral artery aneurysms in the MS group (*p* = 0.022), a higher intake of antiplatelets in the EV group (*p* = 0.016) at t_1_, an unsurprisingly longer duration of MS vs. EV (*p* = 0.004), and a longer mean time spent on mechanical ventilatory support in the EV group than in the pSAH group (EV vs. pSAH *p* = 0.0496; EV vs. MS *p* = 0.864, MS vs. pSAH *p* = 0.065).

### Correlation of neuropsychological performance with CGRP exposure in serum

The self-reported hrQoL perception has been reported previously (22, 23) sum up, our cohort performed significantly worse in several ISR- and SF-36-subscales than the particular population norms, especially at t_1_. Within 6 months, sSAH patients had significantly improved with regard to bodily pain (Pain), physical functioning (Pfi), the physical component summary (PCS), depression, anxiety, general health perceptions (Ghp), and social functioning (Social). Poor self-reported neuropsychological performance [physical SF-36 items: Pain, role limitations because of physical health problems (Rolph), Ghp; ISR scores: total, depression, compulsive-obsessive syndrome] in the subacute phase correlated with a worse outcome on the GOS at discharge. And the HH score correlated positively with all psychological SF-36 item scores [vitality (Vital), Social, role limitations because of emotional problems (Rolem), general mental health (Mhi), mental component summary (MCS)] at t_1_.

**Figure 2:**
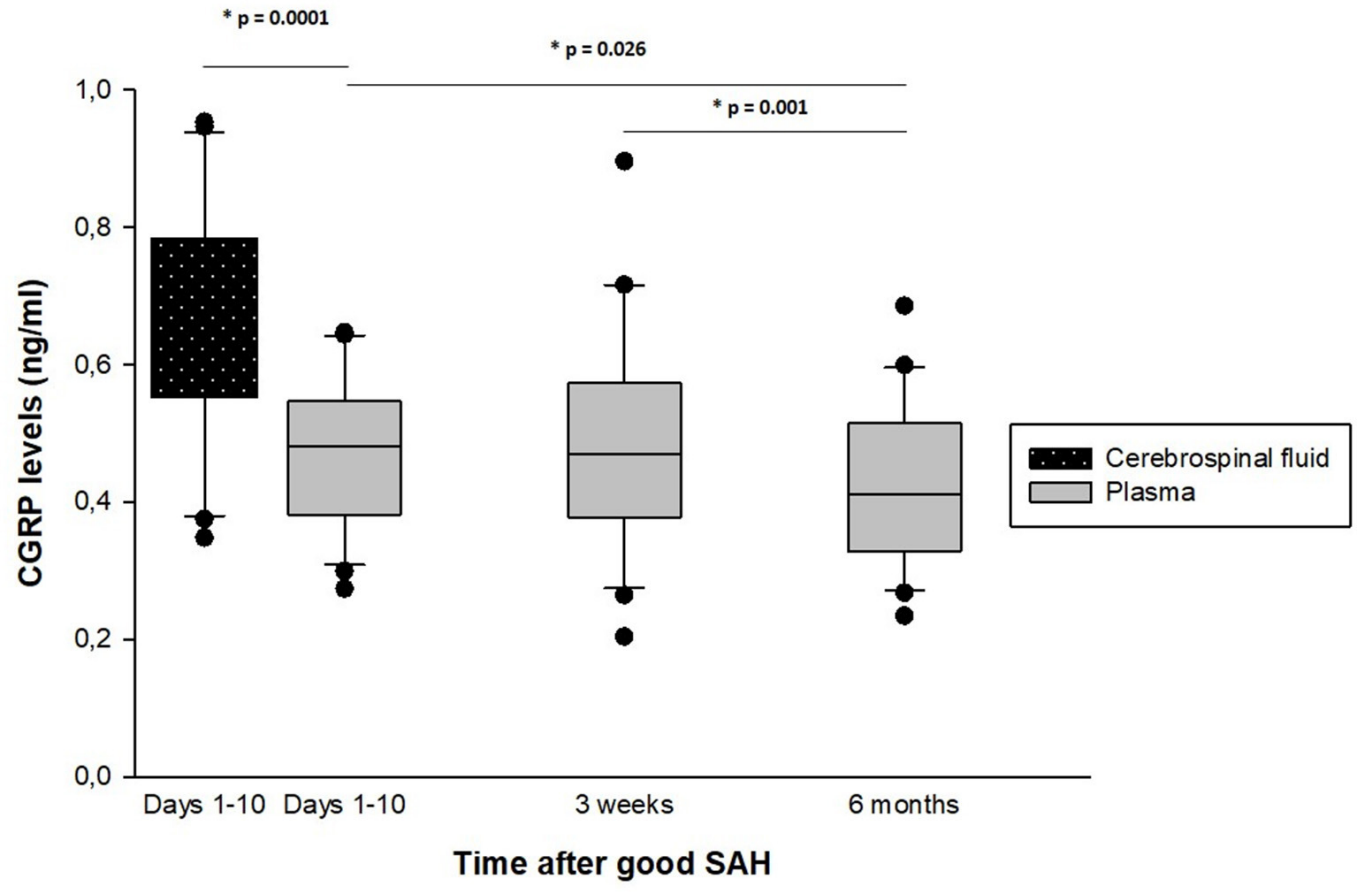
Temporal dynamics of CGRP levels in serum and cerebrospinal fluid after good-grade spontaneous subarachnoid hemorrhage. The time interval (1–10 days, 3 weeks, and 6 months) after good-grade spontaneous subarachnoid hemorrhage, plotted vs. the mean endogenous calcitonin gene-related peptide (CGRP) concentrations in serum. During the first 10 days, the mean CGRP levels were significantly lower in serum than in the cerebrospinal fluid (16). At 6 months, the mean serum CGRP value was significantly lower compared to the first 10 days and compared to 3 weeks.

During the first 10 days, the mean CGRP levels in serum (0.470 ± 0.10 ng/ml) were significantly lower than the previously (16) analyzed mean CGRP values in CSF (0.662 ± 0.173; *p* = 0.0001). The mean serum CGRP levels within the first 10 days did not differ significantly from the values at 3 weeks (0.493 ± 0.163 ng/ml; *p* = 0.304). At 6 months, the mean serum CGRP value (0.429 ± 0.121 ng/ml) was significantly lower compared to 3 weeks (*p* = 0.010) and compared to the first 10 days (*p* = 0.026) (cf. Figure 2).

In female patients, CGRP levels at the 6-month FU were significantly higher than in male patients (0.473 ± 0.121 ng/ml vs. 0.358 ±0.087 ng/ml; *p* = 0.016). Further regression analyses did not reveal any significant correlation between the neuropeptide levels and other patient variables like, for example, the treatment modality.

**Figure 3:**
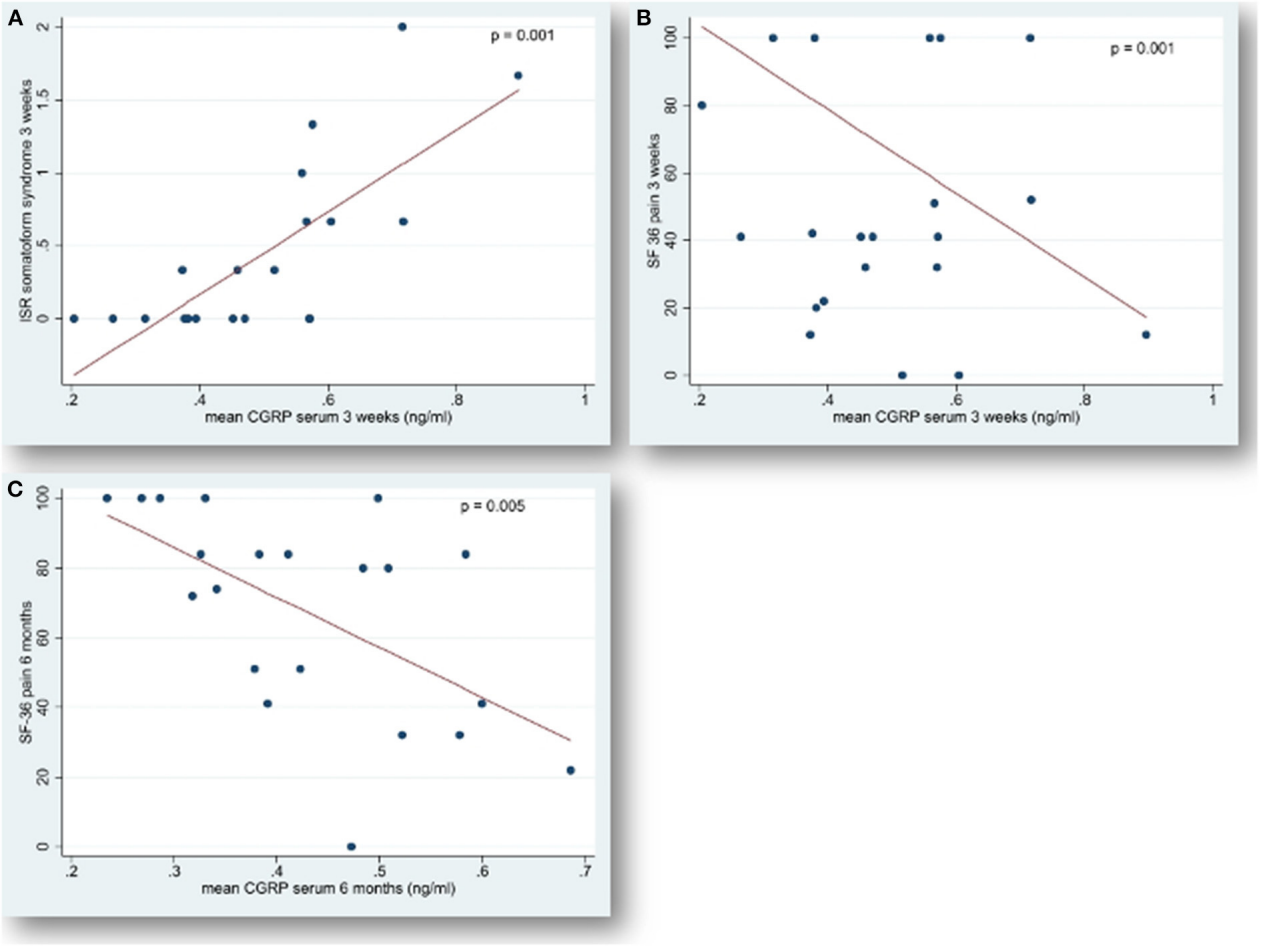
Correlation of serum concentrations of CGRP with “somatoform syndrome” and “pain” after spontaneous subarachnoid hemorrhage. Endogenous calcitonin gene-related peptide (CGRP) levels in serum, measured 6 months (t_2_) after the onset of spontaneous subarachnoid hemorrhage, plotted versus **(A)** somatoform syndrome 3 weeks after the hemorrhage and versus **(B)** pain 3 weeks and **(C)** pain 6 months after ictus. The ICD-10-Symptom-Rating questionnaire (ISR) (30) with 29 items and 6 syndrome scales aims at comprehensively evaluating the severity of psychological disorders. Each syndrome scale ranges from a minimum of 0 (best performance) to a maximum of 4 points with higher scores indicating a more severe symptom burden. The 36-Item Short Form Health Survey (SF-36) (31) is a 36-item generic general health questionnaire yielding scores on 8 health subscales relating to physical and psychological health. These 8 subscales can be summarized in a corresponding physical component summary and a mental component summary. Each item is scored in the range 0 to 100, and a high score defines a more favorable state of health. Items in the same scale are averaged together to create the 8 scale scores. Each dot represents the mean level of serum CGRP in [ng/ml] for each patient, indicating a significant linear correlation (compare regression line) with higher symptom burden (ISR) and with poorer neurobehavioral performance (SF-36). Statistical significance: *p* < 0.05.

Higher mean serum CGRP levels at 3 weeks (*p* = 0.001) and at the 6-month FU (*p* = 0.005) correlated with a significantly poorer performance in the SF-36 item pain, and, at 3 weeks, with a higher ISR symptom burden regarding somatoform syndrome (*p* = 0.001) at t_2_ (cf. Figure 3). The analysis of cognitive test performances is summarized in Table 2.

**Table 2:** Cognitive performance of the cohort (*n* = 21) in the subacute interval after the onset of spontaneous subarachnoid hemorrhage (t_1_) and at 6-month follow-up (t_2_).

**Neuropsychological assessment**	**Test scores [mean ±SD]**		**Difference t_1_vs. t_2_** **[mean ±SD]**	**Paired t-Test t_1_vs. t_2_** **[*p*-value]**
	**t_1_**	**t_2_**		
**ISR scores**				
Depression	1.5 ± 1.2	1.1 ± 0.9	0.4 ± 1.1	0.046^*^
Anxiety	1.3 ± 1.1	0.7 ± 0.9	0.7 ± 0.3	0.019^*^
Compulsive-obsessive	0.8 ± 1.0	0.8 ± 1.0	0.0 ± 1.4	0.480
Somatoform	0.6 ± 0.7	0.4 ± 0.6	0.1 ± 1.0	0.252
Nutrition disorder	0.6 ± 1.0	0.6 ± 0.7	−0.0 ± 1.2	0.523
Supplementary items	0.7 ± 0.5	0.5 ± 0.6	0.2 ± 0.6	0.064
Total	0.9 ± 0.7	0.7 ± 0.6	0.2 ± 0.8	0.098
**SF-36 scores**				
*Physical items*				
Rawhtran	4.5 ± 0.6	2.9 ± 1.4	1.6 ± 1.4	1.000
Pfi	19.5 ± 28.9	72.4 ± 26.3	−52.8 ± 35.5	0.001^*^
Rolph	39.3 ± 43.7	47.6 ± 45.3	−8.3 ± 64.4	0.280
Pain	48.5 ± 34.6	67.2 ± 29.9	−18.7 ± 37.9	0.018^*^
Ghp	56.3 ± 16.8	74.1 ± 18.2	−17.8 ± 19.0	0.001^*^
*Psychological items*				
Vital	51.0 ± 19.7	51.2 ± 20.3	−0.2 ± 24.4	0.482
Social	66.1 ± 26.0	80.4 ± 25.2	−14.3 ± 28.6	0.017^*^
Rolem	61.7 ± 46.2	61.7 ± 45.0	−0.0 ± 58.8	0.500
Mhi	61.1 ± 20.6	68.2 ± 21.7	−7.0 ± 28.6	0.137
PCS	31.0 ± 11.0	46.0 ± 10.0	−13.3 ± 12.7	0.001^*^
MCS	49.1 ± 12.5	47.5 ± 10.7	1.4 ± 12.9	0.677

SD, standard deviation; test t_1_, test in the subacute phase after the onset of bleeding (between day 11 and 35 after subarachnoid hemorrhage); test t_2_, test in the short-term (chronic phase) after treatment at 6-month follow-up; AUC, area under the curve; CGRP, calcitonin gene-related peptide; ISR, ICD-10-Symptom-Rating questionnaire; SF-36, German version of the 36-Item Short Form Health Survey; Rawhtran, health transition item; Pfi, physical functioning; Rolph, role limitations because of physical health problems; Pain, bodily pain; Ghp, general health perceptions; Vital, vitality; Social, social functioning; Rolem, Role limitations because of emotional problems; Mhi, general mental health; PCS, physical component summary; MCS, Mental component summary.

* **Statistical significance: *p*< 0.05**.

## Discussion

Our study characterizes the association of elevated serum CGRP on adverse mid-term hrQoL and burdensome pain after sSAH.

### Reduced hrQoL after sSAH with a focus on pain processing

Compared to other life-threatening medical events, stroke has been proven to be associated with the strongest reductions in hrQoL and functioning status (32). In stroke patients, the physical hrQoL increased after treatment (except for bodily pain), whereas the psychological quality remained low (33). This equals our results in sSAH which indicate a significant improvement in multiple physical items (Pfi, Pain, Ghp, PCS) within 6 months after sSAH, while most psychological items (Compulsive-obsessive syndrome, somatoform syndrome, nutrition disorder, the ISR supplementary items, the ISR total score, Vital, Rolem, Mhi, and MCS) remained stable.

#### hrQoL restrictions after sSAH

A considerable proportion of sSAH survivors with “good recovery” experiences impaired hrQoL for several months up to 24.5 years after ictus or even longer (5, 34). In a current empirical survey (4), one third of the aSAH patients was afflicted with a “post-aSAH syndrome” comprising a self-reported cluster of symptoms like fatigue, cognitive dysfunction (memory, concentration, slowed thought process), emotional problems (anxiety, depression, irritability, and impatience), and somatic problems (headache, sensitivity to sound, and sleep disturbances). The presence of this syndrome–implicating more pain, more pronounced cognitive impairment, and, consecutively, poorer hrQoL–almost invariably kept the patients from returning to work 1 year after the bleeding event (4). This is consistent with our 6-month results (cf. Table 1). Occupational reintegration difficulties and long-term disability after sSAH have a significance extending far beyond socioeconomic terms (8, 34).

Our study emphasizes hrQoL restrictions due to pain conditions. SAH represents a subtype of stroke with a particularly high predisposition to headache in the acute (6) and chronic stage (8, 10). The paucity of analyses available state a higher headache burden in 77% of good-grade SAH patients (35), in 62% of pSAH survivors (10) and 16% of SAH long-term survivors (8) several years after ictus. Most recently, Huckhagel et al. (2) published the first comprehensive data on a significant number of SAH patients with peak rates even reaching up to 40.9% who continued to experience burdensome headaches with a profound negative impact on the hrQoL on average 32.6 months after aSAH. Our study corroborates pain conditions 6 months after ictus. Concordantly, post-stroke headache has been proposed to persist for weeks to months, or even years after the hemorrhage (2, 8, 10). Reduced physical activity not only during the ictal but also during the pain-free interictal phases of migraineurs underlines the intensity-independent disabling effect of headache conditions (36). The SAH headache has been described to be highly refractory and to respond poorly to treatment with standard analgesics (11, 37).

#### Potential determinants for pain conditions and hrQoL restrictions after sSAH

Potential predictor variables, significantly related to chronic headaches after SAH, were nighttime sleep difficulties with daytime sleepiness (8) and correspondingly, fatigue, an acknowledged risk factor for persistent cephalgia after ischemic stroke (38) and a common feature in systemic autoimmune diseases (39). In our study design, patients with autoimmune or systemic diseases were excluded to minimize confounders. Beyond, comorbidities like depression and mood issues may interfere and exacerbate or perpetuate post-stroke headache (38). Patients with a younger mean age at the onset of SAH and patients with a favorable neurological condition as to the WFNS were more prone to suffer from headache at FU (2). All of our sSAH groups developed a significant deterioration in the health transition item (Rawhtran) implicating recurrent headache. It could be argued that good-grade sSAH patients, being aware of a sudden, life-threatening medical event, might experience a psychological traumatization by the onset of sSAH and the intensive care treatment (40), whereas burdened patients without trauma-related memories or with cognitive deficits are at risk of an under-reporting of symptoms and minimizing of hrQoL complaints (41).

When previously (23) analyzing our cohort (with similar EV-MS quota) according to the treatment groups, at 6 month-FU, MS patients experienced less Pain (*p* = 0.040) and yielded significantly better Pfi (*p* = 0.046) than the EV group. Interpreting our results with caution due to the small sample size, we argue that headache might be–at least in part–related to the longer persistence of subarachnoid blood in the basal cisterns causing meningeal irritation. MS affords the opportunity to both obliterate the aneurysm and, compared to EV, to reduce the burden of cisternal blood *via* intraoperative irrigation. The majority of authors dismissed the hypothesis of treatment-specific differences (MS vs. EV) in neuropsychological outcome [cf. (23)] and headache (2) after sSAH in most instances.

#### Pathophysiological considerations

The underlying pathomechanisms contributing to post-sSAH behavioral and cognitive impairment, reduced hrQoL, and associated chronic headache remain to be sufficiently elucidated (1). A diversity of pathophysiological cascades, induced by the hemorrhage and the sharp rise in intracranial pressure itself causing dural stretch as well as the consecutive secondary brain injury, is supposed to be involved (5). Cortical ischemic strokes are more frequently associated with headache than subcortical events (42). Accordingly, it might be argued that SAH affects the subarachnoid space, anatomically a region directly adjacent to the cortex and, thus, sSAH patients tend to be prone to headache. The presence of erythrocytes and blood breakdown products in the subarachnoid space is a potent trigger of meningeal irritation which is further exacerbated by the release of inflammatory cytokines inducing the infiltration of microglia and other immune cells and subsequent pain (11). Beyond, dysfunctions in autoregulatory mechanisms in cerebral perfusion and CV (37), a central sensitization of nociceptive pathways (38), a chronic hyperreactivity of the cerebral vasculature (43), and a stimulation of vascular nerve endings as blood vessels rupture (14, 44) are supposed to aggravate headache.

Pursuing an innovative approach, for the past decade, neuroscientists have increasingly focused on the early identification of valid and easily accessible biomarkers to reliably predict the outcome following sSAH and to design individualized, target-based treatment strategies for improvement of functional outcome and hrQoL (16, 45). Among the molecular genetic, inflammatory, vascular, oxidative stress, and protein biomarkers studied so far, the multifunctional endogenous neuropeptide CGRP appears rather promising (14–16, 44, 46).

### Relevance of CGRP in pain processing after sSAH

The trigeminovascular system is involved in the regulation of the cranial vasculature and represents a key element in the transmission of pain (47). Upon activation in primary headaches, in stroke (48), and in SAH (15, 16, 44, 49), the trigeminovascular system, a vasodilator pathway, antidromically releases the peptide neurotransmitter CGRP (13), hereby increasing the cerebral blood flow (CBF) *via* vasodilation (13) and mediating pain by stimulation of the trigeminus nucleus caudalis, respectively (47). The activation of this (putative defense) system is noted clinically as a marked increase in the cranial venous outflow of CGRP during headache attacks or in SAH, where it was elevated in the CSF as well (14). The psychoactive mediator (17) CGRP has been repeatedly attested a crucial involvement in a variety of neurobehavioral and psycho-affective conditions (18), like in depression, anxiety and learning and memory [cf. (16)], as well as in inflammatory and neuropathic pain (19, 50), and, by pronounced arterial cerebral vasodilation, in migraine (20). Congruently, in our series, elevated CGRP levels in serum at 3 weeks and at 6 months significantly correlated with more pain and, at 3 weeks, with a higher symptom burden regarding somatoform syndrome 6 months after sSAH. We qualify the statement with the note that our utilized set of standardized and validated physical and mental health questionnaires refers to bodily pain in general and is therefore incapable to establish a headache-specific diagnosis. However, in the semi-structured interview during FU assessment recurrent headache was a commonly reported symptom in our series. To what extent CGRP is involved in non-headache conditions has not been clarified yet. A recent review article (19) revealed an association between measured CGRP levels and somatic visceral, inflammatory and neuropathic pain. Upregulated CGRP levels were reported in serum, CSF, synovial and tissue biopsies in patients with degenerative disc disease, osteoarthritis, and temporomandibular joint pain. Furthermore, CGRP was elevated in acute pain conditions and pain after exercise. In somatic pain conditions in particular, CGRP levels correlated with pain. This is in concert with our results as to somatoform syndrome after sSAH. Growing evidence indicates that CGRP facilitates nociceptive transmission and contributes to the development and maintenance of a sensitized, hyperresponsive state of the primary and second-order afferent sensory neurons, thus contributing to peripheral and central sensitization. The maintenance of such a sensitized neuronal condition is believed to be a key player underlying migraine pathophysiology (50). In sSAH, the (potentially adverse) effects of CGRP on pain modulation and transmission have not been explored before. Our data conflicts with the substantiated beneficial cerebroprotective role of (endo- and exogenously administered) CGRP in hemodynamics (14, 15, 46).

Upon nerve stimulation, CGRP is released from its storage vesicles in sympathetic perivascular fibers, which form a particularly dense network around the major arteries of the anterior part of the circle of Willis (14), and in free nerve endings in the *dura mater* (51). Pathophysiologically, it is supposed that, at the moment of aneurysm rupture, these CGRP-containing nerve fibers are affected directly or indirectly–by the stimulation of the vessel's wall or surrounding nociceptors or cortical neuronal depolarization secondary to the hemorrhage or by the blood in the subarachnoid space–initiating activity of the trigeminovascular system and inducing an excessive release of CGRP from the perivascular nerve terminals into CSF and serum (14, 15, 44). *Post mortem* analyses after SAH and early observational studies on the cerebral circulation after experimental SAH confirmed a marked decrease in CGRP immunoreactivity in the perivascular nerve fibers, accordingly [cf. (46)]. To date, our study is the first to provide an insight into the temporal dynamic changes of CGRP in serum after good-grade sSAH. Within the first 10 days and 3 weeks after the onset of hemorrhage, CGRP levels did not differ significantly. In contrast, 6 months thereafter, serum CGRP values had significantly declined. Data on the time course of CGRP in the serum is scarce. The most marked suppression of CGRP immunoreactivity in cerebrovascular nerve fibers was documented during the 7th to 14th day after artificial SAH in dogs with a recovery to normal levels by the 42nd day (52). Hypothetically, the CGRP release is followed by an inhibition of the CGRP reuptake at the nerve-ending terminals. CGRP-receptor complexes are formed, and, possibly after a non-competitive saturation of the extra- and intraluminal CGRP receptors, CGRP levels in CSF and serum finally decrease due to the subsequent depletion of the releasing terminal nerve endings. It has further been speculated that different types of stroke like sSAH may alter the synthesis, metabolism, molecule, or receptors of the neurotransmitter CGRP. In the serum of sSAH patients, peak concentrations of CGRP have been measured after rupture of aneuryms of the middle cerebral artery (14, 44) (during days 4–7 after onset of sSAH), in patients with CV-related ischemia (on day 4), and – as to cerebrovascular manipulation–after endoluminal aneurysm treatment *via* coiling (44) (within days 3–5 after sSAH). In our series, the serum CGRP levels did neither differ regarding the anatomical localization of the ruptured aneurysm, nor regarding the treatment-induced mechanical intra- and extraluminal manipulation of the parent vessel in the aneurysm-securing procedure groups and the control group, respectively. Interestingly, in our cohort without gender bias, the female patients expressed significantly higher serum CGRP levels at the 6-month FU than their male counterparts. Although headaches are prevalent in both sexes and in all age groups, women aged 20 up to 50 years are those who have the highest prevalences in Europe (53).

A remarkable and presumably cerebroprotective hypersecretion of CGRP into serum counterbalancing vasoconstriction was previously detected in sSAH patients with vasospasm-related ischemia (44). In our cohort, exclusively comprising good-grade sSAH patients without any CV-related ischemic complications, the excessive release of CGRP into CSF (15, 16) during the first 10 days after ictus contrasts with the significantly lower CGRP levels in serum. This is all the more noteworthy given that even these smaller amounts of CGRP in serum suffice to induce considerable impairment in pain processing and psychosomatic disorders. The sparse neuropeptide findings in serum presumably result from the diluting effect of the nanomolar concentrations of the ventricular CSF CGRP during its absorption into plasma and its clearance over time. On the other hand, our results in a good-grade sSAH collective contradict previous findings in aSAH patients with CV (14, 44), in whom serum CGRP levels have been measured higher than CGRP levels in CSF. To date, it is unclear which source is the best to (non-invasively) access predictive CGRP values, like, recently, in tear fluid (53) of migraine patients.

After three decades of intense research, drugs targeting the trigeminal sensory neuropeptide CGRP or its receptor and, thus, acting on the trigeminal pain system have been specifically designed (21). It is remarkable that, in migraineurs, all CGRP-targeted therapies (CGRP receptor antagonists/gepants and monoclonal antibodies against CGRP or the CGRP receptor) have proven clinical efficacy, whether in preventing attack onset, targeting acute attacks, or reducing attacks in chronic migraine or frequent episodic migraine (21, 54). To date, it is unknown whether CGRP antagonism may provide a beneficial therapeutic approach for the treatment of chronic pain conditions and CV in sSAH as well.

Scrutinizing the frequency and type of self-reported met and unmet needs of SAH survivors 1–2 years and 3–5 years following hemorrhage, headache, fatigue, concentration, memory, and anxiety were the most commonly described, and a large proportion of needs (> 80%) were detected as unmet (55). The pain conditions, real-world deficits, and the neurobehavioral sequelae of an sSAH are not appropriately addressed by current rehabilitation programs, which still mainly focus on physical rehabilitation. Neuroscientists and clinicians should strive for a standardized screening and early identification of patients at-risk and for non-pharmacologic coping strategies that may be effective in pain management and hrQoL improvement to increase patient resiliency (56). Comprehensive rehabilitation programs imply a multidisciplinary approach (57) with physiotherapy, cognitive behavioral therapy, and biofeedback using relaxation techniques in order to reduce pain conditions; in addition, it is recommended to screen for and treat analgesics overuse (38).

In methodological terms, the main strength of our investigation is the prospective, controlled data collection comprising a throughly selected cohort of a representative neurovascular medium volume center. The study population underwent a meticulous liquid biobanking in combination with a standardized comprehensive hrQoL assessment over a considerable FU period of 6 months. Attributed to the abrupt nature of sSAH, biomarkers are not amenable to baseline data acquisition. Furthermore, the utilized hrQoL questionnaires are not designed to precisely establish pain and headache diagnoses and to specify the psychosomatic symptom burden. A major limitation of our experimental clinical study is the circumscribed sample size demanding a cautious interpretation of our findings. We are aware of the potential bias due to the exclusion of five patients. The results are not applicable for sSAH patients in general since our cohort exclusively encompasses prognostically favorable good-grade individuals with the per-protocol exclusion of non-HC patients.

## Conclusion

Our study reveals the first insight into the potential capacity of endogenous CGRP as a predictive psychoactive biomarker in serum after good-grade sSAH. The present data suggests that patients with elevated CGRP levels in serum suffer from sustained pain conditions and psychosomatic symptoms which in turn seriously affect their hrQoL 6 months after sSAH. This investigation serves as a hypotheses-generating clinical-experimental trial. Given that the neurotransmitter CGRP is a certified core element in migraine, nociceptive processing, and in various neurobehavioral disorders, its pathophysiological relevance in post-sSAH pain seems all the more feasible. Considering our interesting results, we advocate and anticipate further basic and clinical research on this issue to characterize the pain syndromes following sSAH and to elucidate the underlying pathopsychological interactions and pathophysiological mechanisms. In the future, the proven efficacy and safety of the CGRP antagonists and antibodies in migraine therapy might pave the way to establish potential, targeted, preventive, and pharmacotherapeutic treatment options for chronic pain conditions.

## Data availability statement

The raw data supporting the conclusions of this article will be made available by the authors, without undue reservation.

## Ethics statement

The study protocol was approved by the Local Institutional Ethics Committee of the University Medical Center Regensburg (protocol number 06-179). The patients/participants provided their written informed consent to participate in this study. Written informed consent was obtained from the individual(s) for the publication of any potentially identifiable images or data included in this article.

## Author contributions

EB, PS, SB, MP, and E-MS: acquisition of data. EB, K-MS, MP, and FZ: analysis, interpretation of data, and statistical analysis. EB: drafting the article. K-MS and EB: study supervision. K-MS: conception and design and approvement of the final version of the manuscript on behalf of all authors. All authors critically revising the article and review of the submitted version of the manuscript.

## Conflict of interest

The authors declare that the research was conducted in the absence of any commercial or financial relationships that could be construed as a potential conflict of interest.

## Publisher's note

All claims expressed in this article are solely those of the authors and do not necessarily represent those of their affiliated organizations, or those of the publisher, the editors and the reviewers. Any product that may be evaluated in this article, or claim that may be made by its manufacturer, is not guaranteed or endorsed by the publisher.

## Abbreviations

ANOVA: analysis of variance
aSAH: aneurysmal subarachnoid hemorrhage
AUC: area under the curve
CBF: cerebral blood flow
CGRP: calcitonin gene-related peptide
CSF: cerebrospinal fluid
CT (scan): computed tomography (scan)
CV: cerebral vasospasm
DIND: delayed ischemic neurological deficit
DSA: digital subtraction angiography
EIA: enzyme immunoassay
EV: endovascular aneurysm occlusion
EVD: external ventricular drain
FU: 6-month follow-up
GCS: Glasgow Coma Scale
Ghp: general health perceptions
GOS: Glasgow Outcome Scale
h: hour/s
HH: Hunt and Hess
hrQoL: health-related quality of life
ICU: intensive care unit
ISR: ICD-10-Symptom-Rating questionnaire
MCS: mental component summary
Mhi: general mental health
min: minute/s
min-max: minimum - maximum
ml: milliliter
mRS: modified Ranking Scale
MS, microsurgical clipping: microsurgical aneurysm occlusion
ng: nanograms
no., [n]: number
NPY: Neuropeptide Y
p: p-value
Pain: bodily pain
PCS: physical component summary
Pfi: physical functioning
pSAH: perimesencephalic subarachnoid hemorrhage
PTSD: posttraumatic stress disorder
Rawhtran: health transition item
Rolem: role limitations because of emotional problems
Rolph: role limitations due to physical health problems
SAH: subarachnoid hemorrhage
SD: standard deviation
SF-36: 36-Item Short Form Health Survey
Social: social functioning
sSAH: spontaneous subarachnoid hemorrhage
TCD: transcranial Doppler ultrasound
(test) t_1_: test in the subacute phase after the onset of bleeding (between day 11 to 35 after subarachnoid hemorrhage)
(test) t_2_: test in the short-term (chronic phase) after treatment at 6-month follow-up
test t_1_ - t_2_: intergroup development from t_1_ to t_2_
Vital: vitality
vs.: versus
WFNS: World Federation of Neurological Surgeons.
